# Tetra­kis(azido-κ*N*)(di-2-pyridyl­amine-κ^2^ *N* ^2^,*N* ^2′^)platinum(IV)

**DOI:** 10.1107/S1600536812011178

**Published:** 2012-03-21

**Authors:** Kwang Ha

**Affiliations:** a School of Applied Chemical Engineering, The Research Institute of Catalysis, Chonnam National University, Gwangju 500-757, Republic of Korea

## Abstract

In the title complex, [Pt(N_3_)_4_(C_10_H_9_N_3_)], the Pt^IV^ ion is six-coordinated in a slightly distorted octa­hedral environment by the two pyridine N atoms of the chelating di-2-pyridyl­amine (dpa) ligand and four N atoms from four azide anions. The dpa ligand is not planar, the dihedral angle between the pyridine rings being 20.0 (3)°. The azide ligands are slightly bent [N—N—N = 173.5 (8)–175.1 (8)°]. In the crystal, the complex mol­ecules are connected by N—H⋯N hydrogen bonds, forming a chain along the *b* axis. An inter­molecular π–π inter­action between the chains is also present, the ring centroid–centroid distance being 3.713 (4) Å.

## Related literature

For the crystal structure of the related chlorido Pt^IV^ complex [PtCl_4_(dpa)], see: Ha (2011 ▶).
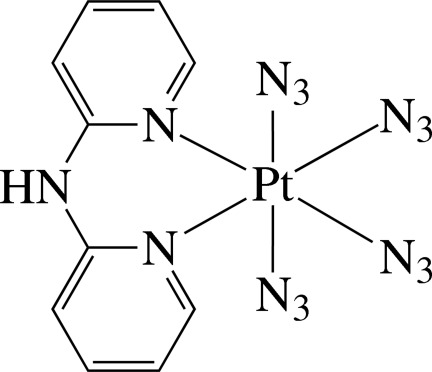


## Experimental

### 

#### Crystal data

[Pt(N_3_)_4_(C_10_H_9_N_3_)]
*M* *_r_* = 534.41
Monoclinic, 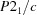

*a* = 7.0057 (4) Å
*b* = 14.7685 (9) Å
*c* = 14.9633 (9) Å
β = 98.118 (1)°
*V* = 1532.64 (16) Å^3^
*Z* = 4
Mo *K*α radiation
μ = 9.19 mm^−1^
*T* = 200 K
0.18 × 0.07 × 0.06 mm

#### Data collection

Bruker SMART 1000 CCD diffractometer
Absorption correction: multi-scan (*SADABS*; Bruker, 2000 ▶) *T*_min_ = 0.420, *T*_max_ = 0.576
9422 measured reflections
3004 independent reflections
2132 reflections with *I* > 2σ(*I*)
*R*_int_ = 0.063

#### Refinement

*R*[*F* ^2^ > 2σ(*F* ^2^)] = 0.036
*wR*(*F* ^2^) = 0.077
*S* = 0.94
3004 reflections
235 parameters
H-atom parameters constrained
Δρ_max_ = 2.25 e Å^−3^
Δρ_min_ = −0.86 e Å^−3^

### 

Data collection: *SMART* (Bruker, 2000 ▶); cell refinement: *SAINT* (Bruker, 2000 ▶); data reduction: *SAINT*; program(s) used to solve structure: *SHELXS97* (Sheldrick, 2008 ▶); program(s) used to refine structure: *SHELXL97* (Sheldrick, 2008 ▶); molecular graphics: *ORTEP-3* (Farrugia, 1997 ▶) and *PLATON* (Spek, 2009 ▶); software used to prepare material for publication: *SHELXL97*.

## Supplementary Material

Crystal structure: contains datablock(s) global, I. DOI: 10.1107/S1600536812011178/is5092sup1.cif

Structure factors: contains datablock(s) I. DOI: 10.1107/S1600536812011178/is5092Isup2.hkl

Additional supplementary materials:  crystallographic information; 3D view; checkCIF report

## Figures and Tables

**Table 1:** Selected bond lengths (Å)

Pt1—N4	2.029 (7)
Pt1—N7	2.030 (7)
Pt1—N13	2.057 (6)
Pt1—N1	2.061 (6)
Pt1—N3	2.067 (6)
Pt1—N10	2.076 (6)

**Table 2:** Hydrogen-bond geometry (Å, °)

*D*—H⋯*A*	*D*—H	H⋯*A*	*D*⋯*A*	*D*—H⋯*A*
N2—H2*N*⋯N10^i^	0.92	2.13	3.031 (9)	167
N2—H2*N*⋯N11^i^	0.92	2.60	3.381 (9)	143

Symmetry code: (i) 
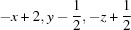
.

**Fig. 1.**
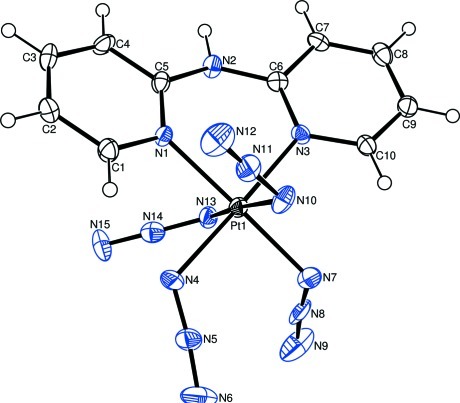
A structure detail of the title complex, with displacement ellipsoids drawn at the 40% probability level for non-H atoms.

**Fig. 2.**
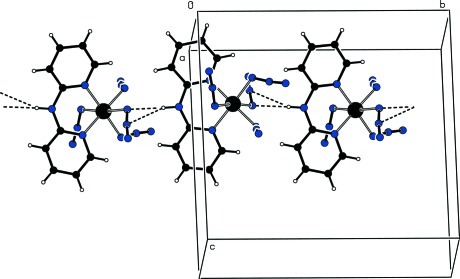
A partial view of the unit-cell contents of the title complex. Intermolecular N—H···N hydrogen-bond interactions are drawn with dashed lines.

## supplementary crystallographic information

### Comment

Crystal structure of the related chlorido Pt^IV^ complex, [PtCl_4_(dpa)] (dpa = di-2-pyridylamine, C_10_H_9_N_3_), has been reported previously (Ha, 2011).

In the title complex, [Pt(N_3_)_4_(dpa)], the Pt^IV^ ion is six-coordinated in a slightly distorted octahedral environment by the two pyridine N atoms of the chelating dpa ligand and four N atoms from four azide anions (Fig. 1). In the crystal structure, the dpa ligand is not planar. The dihedral angle between the least-squares planes of the pyridine rings is 20.0 (3)°. The Pt—N(dpa) and Pt—N(azide) bond lengths are nearly equivalent [Pt—N: 2.029 (7)–2.076 (6) Å] (Table 1). The azido ligands are slightly bent with the bond angles of <N4—N5—N6 = 174.1 (9)°, <N7—N8—N9 = 173.5 (8)°, <N10—N11—N12 = 174.3 (8)° and <N13—N14—N15 = 175.1 (8)°. But, the N—N bond lengths of the ligands are almost equal [N—N: 1.129 (9)–1.236 (9) Å]. The complex molecules are stacked in columns along the *a* axis and are connected by intermolecular N—H···N hydrogen bonds, forming chains along the *b* axis (Fig. 2 and Table 2). Along the *b* axis, successive chains stack in opposite directions. An intermolecular π–π interaction between the pyridine rings is also present, the ring centroid-centroid distance being 3.713 (4) Å.

### Experimental

To a solution of Na_2_PtCl_6_.6H_2_O (0.1684 g, 0.300 mmol) in MeOH (30 ml) were added NaN_3_ (0.2129 g, 3.275 mmol) and di-2-pyridylamine (0.1046 g, 0.611 mmol), and the mixture was refluxed for 5 h. The formed precipitate was separated by filtration and washed with H_2_O and MeOH, and dried at 50 °C, to give a yellow powder (0.0687 g). Crystals suitable for X-ray analysis were obtained by slow evaporation from an acetone solution.

### Refinement

Carbon-bound H atoms were positioned geometrically and allowed to ride on their respective parent atoms [C—H = 0.95 Å and *U*_iso_(H) = 1.2*U*_eq_(C)]. Nitrogen-bound H atom was located in a difference Fourier map and then allowed to ride on its parent atom in the final cycles of refinement with N—H = 0.92 Å and *U*_iso_(H) = 1.5 *U*_eq_(N). The highest peak (2.25 eÅ^-3^) and the deepest hole (-0.86 eÅ^-3^) in the difference Fourier map are located 1.25 Å and 1.09 Å from the atoms N4 and C10, respectively.

### Crystal data

**Table:**

[Pt(N_3_)_4_(C_10_H_9_N_3_)]	*F*(000) = 1008
*M**_r_* = 534.41	*D*_x_ = 2.316 Mg m^−^^3^
Monoclinic, *P*2_1_/*c*	Mo *K*α radiation, λ = 0.71073 Å
Hall symbol: -P 2ybc	Cell parameters from 3131 reflections
*a* = 7.0057 (4) Å	θ = 2.8–25.9°
*b* = 14.7685 (9) Å	µ = 9.19 mm^−^^1^
*c* = 14.9633 (9) Å	*T* = 200 K
β = 98.118 (1)°	Block, yellow
*V* = 1532.64 (16) Å^3^	0.18 × 0.07 × 0.06 mm
*Z* = 4	

### Data collection

**Table:**

Bruker SMART 1000 CCD diffractometer	3004 independent reflections
Radiation source: fine-focus sealed tube	2132 reflections with *I* > 2σ(*I*)
Graphite monochromator	*R*_int_ = 0.063
φ and ω scans	θ_max_ = 26.0°, θ_min_ = 2.0°
Absorption correction: multi-scan (*SADABS*; Bruker, 2000)	*h* = −8→8
*T*_min_ = 0.420, *T*_max_ = 0.576	*k* = −18→17
9422 measured reflections	*l* = −18→16

### Refinement

**Table:**

Refinement on *F*^2^	Primary atom site location: structure-invariant direct methods
Least-squares matrix: full	Secondary atom site location: difference Fourier map
*R*[*F*^2^ > 2σ(*F*^2^)] = 0.036	Hydrogen site location: inferred from neighbouring sites
*wR*(*F*^2^) = 0.077	H-atom parameters constrained
*S* = 0.94	*w* = 1/[σ^2^(*F*_o_^2^) + (0.0288*P*)^2^] where *P* = (*F*_o_^2^ + 2*F*_c_^2^)/3
3004 reflections	(Δ/σ)_max_ < 0.001
235 parameters	Δρ_max_ = 2.25 e Å^−^^3^
0 restraints	Δρ_min_ = −0.86 e Å^−^^3^

### Special details

**Table:**

Geometry. All e.s.d.'s (except the e.s.d. in the dihedral angle between two l.s. planes) are estimated using the full covariance matrix. The cell e.s.d.'s are taken into account individually in the estimation of e.s.d.'s in distances, angles and torsion angles; correlations between e.s.d.'s in cell parameters are only used when they are defined by crystal symmetry. An approximate (isotropic) treatment of cell e.s.d.'s is used for estimating e.s.d.'s involving l.s. planes.
Refinement. Refinement of *F*^2^ against ALL reflections. The weighted *R*-factor *wR* and goodness of fit *S* are based on *F*^2^, conventional *R*-factors *R* are based on *F*, with *F* set to zero for negative *F*^2^. The threshold expression of *F*^2^ > σ(*F*^2^) is used only for calculating *R*-factors(gt) *etc*. and is not relevant to the choice of reflections for refinement. *R*-factors based on *F*^2^ are statistically about twice as large as those based on *F*, and *R*- factors based on ALL data will be even larger.

### Fractional atomic coordinates and isotropic or equivalent isotropic displacement parameters (Å^2^)

**Table:**

	*x*	*y*	*z*	*U*_iso_*/*U*_eq_	
Pt1	0.81972 (4)	0.15708 (2)	0.266198 (19)	0.02164 (11)	
N1	0.8308 (9)	0.0693 (4)	0.1599 (4)	0.0190 (14)	
N2	0.9443 (9)	−0.0523 (4)	0.2561 (4)	0.0229 (15)	
H2N	0.9692	−0.1135	0.2562	0.034*	
N3	1.0214 (9)	0.0791 (4)	0.3460 (4)	0.0200 (14)	
C1	0.7670 (11)	0.0998 (5)	0.0743 (5)	0.0256 (19)	
H1	0.7444	0.1627	0.0644	0.031*	
C2	0.7353 (11)	0.0410 (6)	0.0027 (5)	0.0279 (19)	
H2	0.6910	0.0628	−0.0563	0.033*	
C3	0.7682 (12)	−0.0494 (6)	0.0174 (5)	0.032 (2)	
H3	0.7438	−0.0911	−0.0313	0.038*	
C4	0.8357 (12)	−0.0795 (5)	0.1014 (5)	0.0271 (19)	
H4	0.8587	−0.1423	0.1115	0.033*	
C5	0.8713 (11)	−0.0191 (5)	0.1725 (5)	0.0211 (17)	
C6	1.0400 (11)	−0.0099 (5)	0.3320 (5)	0.0201 (17)	
C7	1.1569 (11)	−0.0630 (5)	0.3944 (5)	0.0229 (18)	
H7	1.1680	−0.1262	0.3847	0.028*	
C8	1.2555 (11)	−0.0231 (6)	0.4697 (5)	0.0278 (19)	
H8	1.3342	−0.0587	0.5132	0.033*	
C9	1.2400 (11)	0.0690 (6)	0.4821 (5)	0.028 (2)	
H9	1.3085	0.0978	0.5337	0.034*	
C10	1.1251 (11)	0.1176 (6)	0.4191 (5)	0.0232 (18)	
H10	1.1172	0.1813	0.4267	0.028*	
N4	0.6200 (11)	0.2306 (5)	0.1855 (4)	0.0369 (19)	
N5	0.5449 (10)	0.2945 (5)	0.2159 (4)	0.0302 (16)	
N6	0.4660 (13)	0.3556 (6)	0.2374 (6)	0.063 (3)	
N7	0.8045 (10)	0.2450 (5)	0.3692 (5)	0.0319 (17)	
N8	0.6586 (12)	0.2401 (5)	0.4068 (4)	0.0353 (19)	
N9	0.5310 (14)	0.2423 (6)	0.4464 (6)	0.059 (3)	
N10	1.0370 (10)	0.2427 (4)	0.2364 (4)	0.0281 (17)	
N11	1.0858 (10)	0.2358 (4)	0.1613 (5)	0.0306 (17)	
N12	1.1381 (12)	0.2365 (5)	0.0918 (5)	0.041 (2)	
N13	0.6117 (10)	0.0768 (4)	0.3100 (4)	0.0247 (15)	
N14	0.4674 (10)	0.0646 (4)	0.2548 (4)	0.0281 (17)	
N15	0.3276 (11)	0.0487 (5)	0.2071 (5)	0.043 (2)	

### Atomic displacement parameters (Å^2^)

**Table:**

	*U*^11^	*U*^22^	*U*^33^	*U*^12^	*U*^13^	*U*^23^
Pt1	0.02640 (18)	0.01631 (17)	0.02080 (17)	0.00107 (15)	−0.00153 (12)	−0.00106 (14)
N1	0.024 (4)	0.016 (4)	0.016 (4)	−0.002 (3)	0.001 (3)	−0.001 (3)
N2	0.035 (4)	0.013 (4)	0.019 (4)	−0.001 (3)	−0.003 (3)	0.005 (3)
N3	0.020 (4)	0.019 (4)	0.019 (4)	0.002 (3)	−0.001 (3)	0.003 (3)
C1	0.031 (5)	0.024 (5)	0.020 (5)	0.001 (4)	−0.001 (4)	0.002 (3)
C2	0.026 (5)	0.033 (5)	0.023 (5)	0.002 (4)	−0.003 (4)	0.001 (4)
C3	0.038 (5)	0.039 (6)	0.017 (4)	−0.001 (4)	−0.004 (4)	−0.007 (4)
C4	0.038 (5)	0.016 (5)	0.027 (5)	0.003 (4)	0.003 (4)	−0.006 (3)
C5	0.021 (4)	0.026 (5)	0.016 (4)	−0.004 (4)	0.004 (3)	−0.002 (3)
C6	0.018 (4)	0.020 (5)	0.024 (4)	−0.004 (3)	0.007 (3)	0.002 (3)
C7	0.018 (4)	0.021 (5)	0.030 (5)	0.003 (3)	0.003 (4)	0.005 (3)
C8	0.022 (4)	0.041 (6)	0.020 (4)	0.000 (4)	0.003 (4)	0.004 (4)
C9	0.020 (4)	0.038 (6)	0.027 (5)	0.000 (4)	0.004 (4)	−0.009 (4)
C10	0.021 (4)	0.024 (5)	0.023 (5)	0.002 (4)	−0.002 (4)	0.001 (3)
N4	0.044 (5)	0.033 (5)	0.029 (4)	0.022 (4)	−0.010 (4)	−0.002 (3)
N5	0.035 (4)	0.020 (4)	0.035 (4)	0.003 (4)	0.002 (3)	0.004 (3)
N6	0.070 (7)	0.049 (6)	0.067 (6)	0.038 (5)	0.000 (5)	−0.005 (5)
N7	0.029 (4)	0.029 (4)	0.037 (4)	0.002 (3)	0.004 (4)	−0.009 (3)
N8	0.059 (6)	0.024 (4)	0.022 (4)	−0.001 (4)	0.001 (4)	−0.013 (3)
N9	0.071 (7)	0.049 (6)	0.063 (6)	−0.022 (5)	0.038 (5)	−0.025 (4)
N10	0.039 (4)	0.018 (4)	0.027 (4)	−0.011 (3)	0.005 (3)	−0.005 (3)
N11	0.034 (4)	0.015 (4)	0.041 (5)	−0.006 (3)	−0.003 (4)	−0.002 (3)
N12	0.056 (6)	0.030 (5)	0.040 (5)	−0.007 (4)	0.014 (4)	0.001 (4)
N13	0.029 (4)	0.020 (4)	0.024 (4)	−0.002 (3)	−0.003 (3)	0.000 (3)
N14	0.031 (4)	0.028 (4)	0.027 (4)	0.003 (3)	0.011 (4)	0.003 (3)
N15	0.031 (5)	0.062 (6)	0.030 (5)	−0.005 (4)	−0.011 (4)	0.002 (4)

### Geometric parameters (Å, º)

**Table:**

Pt1—N4	2.029 (7)	C4—C5	1.383 (10)
Pt1—N7	2.030 (7)	C4—H4	0.9500
Pt1—N13	2.057 (6)	C6—C7	1.393 (10)
Pt1—N1	2.061 (6)	C7—C8	1.369 (11)
Pt1—N3	2.067 (6)	C7—H7	0.9500
Pt1—N10	2.076 (6)	C8—C9	1.379 (11)
N1—C5	1.344 (9)	C8—H8	0.9500
N1—C1	1.372 (9)	C9—C10	1.356 (11)
N2—C5	1.373 (9)	C9—H9	0.9500
N2—C6	1.385 (9)	C10—H10	0.9500
N2—H2N	0.9200	N4—N5	1.200 (9)
N3—C6	1.340 (9)	N5—N6	1.129 (9)
N3—C10	1.350 (9)	N7—N8	1.236 (9)
C1—C2	1.373 (10)	N8—N9	1.141 (10)
C1—H1	0.9500	N10—N11	1.223 (9)
C2—C3	1.367 (11)	N11—N12	1.151 (9)
C2—H2	0.9500	N13—N14	1.225 (9)
C3—C4	1.353 (11)	N14—N15	1.152 (9)
C3—H3	0.9500		
			
N4—Pt1—N7	90.2 (3)	C4—C3—H3	120.1
N4—Pt1—N13	92.2 (3)	C2—C3—H3	120.1
N7—Pt1—N13	90.6 (3)	C3—C4—C5	120.3 (8)
N4—Pt1—N1	88.6 (3)	C3—C4—H4	119.9
N7—Pt1—N1	178.8 (3)	C5—C4—H4	119.9
N13—Pt1—N1	89.4 (2)	N1—C5—N2	121.2 (6)
N4—Pt1—N3	178.4 (3)	N1—C5—C4	120.5 (7)
N7—Pt1—N3	91.3 (3)	N2—C5—C4	118.3 (7)
N13—Pt1—N3	87.3 (2)	N3—C6—N2	121.7 (7)
N1—Pt1—N3	89.9 (2)	N3—C6—C7	120.5 (7)
N4—Pt1—N10	90.6 (3)	N2—C6—C7	117.8 (7)
N7—Pt1—N10	83.8 (3)	C8—C7—C6	119.4 (8)
N13—Pt1—N10	173.8 (2)	C8—C7—H7	120.3
N1—Pt1—N10	96.2 (2)	C6—C7—H7	120.3
N3—Pt1—N10	90.1 (3)	C7—C8—C9	119.7 (8)
C5—N1—C1	119.0 (6)	C7—C8—H8	120.2
C5—N1—Pt1	122.3 (5)	C9—C8—H8	120.2
C1—N1—Pt1	118.1 (5)	C10—C9—C8	118.6 (8)
C5—N2—C6	131.3 (6)	C10—C9—H9	120.7
C5—N2—H2N	113.5	C8—C9—H9	120.7
C6—N2—H2N	111.9	N3—C10—C9	122.6 (8)
C6—N3—C10	119.1 (6)	N3—C10—H10	118.7
C6—N3—Pt1	122.0 (5)	C9—C10—H10	118.7
C10—N3—Pt1	118.6 (5)	N5—N4—Pt1	119.8 (6)
N1—C1—C2	121.0 (8)	N6—N5—N4	174.1 (9)
N1—C1—H1	119.5	N8—N7—Pt1	116.3 (5)
C2—C1—H1	119.5	N9—N8—N7	173.5 (8)
C3—C2—C1	119.2 (8)	N11—N10—Pt1	117.1 (5)
C3—C2—H2	120.4	N12—N11—N10	174.3 (8)
C1—C2—H2	120.4	N14—N13—Pt1	115.1 (5)
C4—C3—C2	119.9 (8)	N15—N14—N13	175.1 (8)
			
N4—Pt1—N1—C5	−149.7 (6)	C10—N3—C6—N2	−176.9 (6)
N13—Pt1—N1—C5	−57.5 (6)	Pt1—N3—C6—N2	9.2 (9)
N3—Pt1—N1—C5	29.7 (6)	C10—N3—C6—C7	3.3 (10)
N10—Pt1—N1—C5	119.8 (6)	Pt1—N3—C6—C7	−170.6 (5)
N4—Pt1—N1—C1	20.5 (6)	C5—N2—C6—N3	23.2 (12)
N13—Pt1—N1—C1	112.7 (6)	C5—N2—C6—C7	−157.0 (7)
N3—Pt1—N1—C1	−160.1 (5)	N3—C6—C7—C8	−1.0 (11)
N10—Pt1—N1—C1	−70.0 (6)	N2—C6—C7—C8	179.2 (6)
N7—Pt1—N3—C6	152.3 (6)	C6—C7—C8—C9	−0.9 (11)
N13—Pt1—N3—C6	61.7 (6)	C7—C8—C9—C10	0.6 (11)
N1—Pt1—N3—C6	−27.7 (6)	C6—N3—C10—C9	−3.7 (11)
N10—Pt1—N3—C6	−123.9 (6)	Pt1—N3—C10—C9	170.4 (6)
N7—Pt1—N3—C10	−21.7 (6)	C8—C9—C10—N3	1.8 (12)
N13—Pt1—N3—C10	−112.2 (6)	N7—Pt1—N4—N5	−4.3 (7)
N1—Pt1—N3—C10	158.4 (5)	N13—Pt1—N4—N5	86.4 (7)
N10—Pt1—N3—C10	62.1 (5)	N1—Pt1—N4—N5	175.7 (7)
C5—N1—C1—C2	3.2 (11)	N10—Pt1—N4—N5	−88.1 (7)
Pt1—N1—C1—C2	−167.4 (6)	N4—Pt1—N7—N8	74.2 (6)
N1—C1—C2—C3	0.0 (12)	N13—Pt1—N7—N8	−18.0 (6)
C1—C2—C3—C4	−1.6 (12)	N3—Pt1—N7—N8	−105.3 (6)
C2—C3—C4—C5	0.1 (12)	N10—Pt1—N7—N8	164.8 (6)
C1—N1—C5—N2	176.9 (7)	N4—Pt1—N10—N11	−73.8 (6)
Pt1—N1—C5—N2	−13.0 (9)	N7—Pt1—N10—N11	−164.0 (6)
C1—N1—C5—C4	−4.7 (10)	N1—Pt1—N10—N11	14.8 (6)
Pt1—N1—C5—C4	165.4 (6)	N3—Pt1—N10—N11	104.7 (6)
C6—N2—C5—N1	−21.0 (12)	N4—Pt1—N13—N14	31.4 (6)
C6—N2—C5—C4	160.5 (7)	N7—Pt1—N13—N14	121.6 (6)
C3—C4—C5—N1	3.2 (12)	N1—Pt1—N13—N14	−57.2 (6)
C3—C4—C5—N2	−178.4 (7)	N3—Pt1—N13—N14	−147.1 (6)

### Hydrogen-bond geometry (Å, º)

**Table:**

*D*—H···*A*	*D*—H	H···*A*	*D*···*A*	*D*—H···*A*
N2—H2*N*···N10^i^	0.92	2.13	3.031 (9)	167
N2—H2*N*···N11^i^	0.92	2.60	3.381 (9)	143

Symmetry code: (i) −*x*+2, *y*−1/2, −*z*+1/2.
